# A household case evidences shorter shedding of SARS-CoV-2 in naturally infected cats compared to their human owners

**DOI:** 10.1080/22221751.2020.1863132

**Published:** 2021-03-05

**Authors:** Víctor Neira, Bárbara Brito, Belén Agüero, Felipe Berrios, Valentina Valdés, Alberto Gutierrez, Naomi Ariyama, Patricio Espinoza, Patricio Retamal, Edward C. Holmes, Ana S. Gonzalez-Reiche, Zenab Khan, Adriana van de Guchte, Jayeeta Dutta, Lisa Miorin, Thomas Kehrer, Nicolás Galarce, Leonardo I. Almonacid, Jorge Levican, Harm van Bakel, Adolfo García-Sastre, Rafael A. Medina

**Affiliations:** a Facultad de Ciencias Veterinarias y Pecuarias, Departamento de Medicina Preventiva, Universidad de Chile, Santiago, Chile; b The three institute – University of Technology Sydney, Sydney, Australia; c School of Life and Environmental Sciences and School of Medical Sciences, Marie Bashir Institute for Infectious Diseases and Biosecurity, The University of Sydney, Sydney, Australia; d Department of Genetics and Genomic Sciences, Icahn School of Medicine at Mount Sinai, New York, NY, USA; e Department of Microbiology, Icahn School of Medicine at Mount Sinai, New York, NY, USA; f Global Health and Emerging Pathogens Institute, Icahn School of Medicine at Mount Sinai, New York, NY, USA; g Departamento de Enfermedades Infecciosas e Inmunología Pediátrica, Escuela de Medicina, Pontificia Universidad Católica de Chile, Santiago, Chile; h Icahn Institute for Data Science and Genomic Technology, Icahn School of Medicine at Mount Sinai, New York, NY, USA; i Department of Medicine, Division of Infectious Diseases, Icahn School of Medicine at Mount Sinai, New York, NY, USA; j The Tisch Cancer Institute, Icahn School of Medicine at Mount Sinai, New York, NY, USA

**Keywords:** SARS-CoV-2, natural infection, domestic cats, households, viral shedding

## Abstract

Severe acute respiratory syndrome coronavirus 2 (SARS-CoV-2) has been detected in domestic and wild cats. However, little is known about natural viral infections of domestic cats, although their importance for modelling disease spread, informing strategies for managing positive human-animal relationships and disease prevention. Here, we describe the SARS-CoV-2 infection in a household of two human adults and sibling cats (one male and two females) using real-time RT–PCR, an ELISA test, viral sequencing, and virus isolation. On May 5th, 2020, the cat-owners tested positive for SARS-CoV-2. Two days later, the male cat showed mild respiratory symptoms and tested positive. Four days after the male cat, the two female cats became positive, asymptomatically. Also, one human and one cat showed antibodies against SARS-CoV-2. All cats excreted detectable SARS-CoV-2 RNA for a shorter duration than humans and viral sequences analysis confirmed human-to-cat transmission. We could not determine if cat-to-cat transmission also occurred.

## Introduction

In December 2019, severe acute respiratory syndrome-related coronavirus 2 (SARS-CoV-2) emerged as a new human pathogen in Wuhan, Hubei province, China, likely due to a cross-species transmission event between humans and an unknown animal reservoir [1]. Since then, SARS-CoV-2 has been efficiently transmitted between humans, causing a pandemic with devastating consequences for human health, societies and economies globally [2].

There is also now a growing body of data indicating that the virus is able to infect companion animals and other animal species [1,3,4]. To date, naturally occurring cases officially reported to the World Organization for Animal Health (OIE) and to the United States Department of Agriculture include 14 cases in dogs in the US, Hong Kong, and Japan, and 25 cases in cats in the US, Hong Kong, France, Belgium, Germany, Russia, Spain and the UK [5]. Additionally, cases in farmed mink from the Netherlands, Spain, US, and Denmark, as well as tigers and lions from a New York zoo in the US, have also been reported [5–7]. Dogs that have been experimentally infected show low viral loads and do not observably transmit SARS-CoV-2 between dogs [1]. Among domestic animals, natural infection and experimental data suggest that felids and mustelids are more susceptible to SARS-CoV-2 infection than canines [5, 8–10]. Companion animals are considered as protective factors against loneliness and as a source of social and emotional support for people during difficult situations or disease [11–13].

Understanding how and when SARS-CoV-2 is transmitted in domestic animals is critical for informing decisions for control and prevention of infections. Previous studies of SARS-CoV-2 transmission in cats have been in laboratory settings [9]. Cats experimentally infected with SARS-CoV-2 can efficiently replicate and transmit the virus to other cats housed in adjacent cages, suggesting respiratory transmission. In these species, the virus replicates mostly in the upper airways. Juvenile compared to sub-adult cats have more severe outcomes [8,9,14]. However, nothing is known about patterns of viral transmission under natural conditions in households in which humans inhabits with companion cats. Key questions involve determining the dynamics of virus infection including the duration of viral excretion.

Beginning in May 2020 we conducted active surveillance in Santiago City, Chile, to determine the potential infection of companion animals in households in which the humans had tested positive for SARS-CoV-2. At that time, Santiago was experiencing daily reports of >3000 SARS-CoV-2 human cases. From a survey of 13 households, we found two containing companion animals that tested positive for SARS-CoV-2. One household had two cats, one of which presented antibodies to SARS-CoV-2. The other household was home to three 10-year-old sibling cats: all three were identified as having a SARS-CoV-2-positive infection. Herein, we describe a case-study of naturally occurring SARS-CoV-2 infection based on the three-cat household that also included two human adults aged 33 and 63 years who were both positive for SARS-CoV-2. The data generated provide a better understanding of infection dynamics in cats, one of the most important SARS-CoV-2 animal natural hosts documented to date.

## Materials and methods

### Case description and specimen collection

Between May and September 2020, we collected animal companion samples from 12 households with confirmed human SARS-CoV-2 cases in Santiago City, Chile. These samples were from 17 cats and 10 dogs (Supplementary Table 1). Of the 12 households sampled, two presented animals positive for SARS-CoV-2. In one household, one of two cats had SARS-CoV-2 antibodies measured by ELISA. In the other household, three out of three cats were positive for SARS-CoV-2 by rtRT-PCR. We followed the second-mentioned household for 40 days to examine SARS-CoV-2 infection dynamics. This case is described below.

On May 5th, a COVID-19 diagnosis was confirmed by SARS-CoV-2 rtRT-PCR for the humans in the studied household – a 33-years-old male clinically healthy (human 1) and a 63-year-old female with asthma, hypertension, obesity, and osteoarthritis (human 2). The residents owned three 10-years-old sibling cats, 1 male (cat 1) and 2 females (cat 2 & 3). Of the three cats, cat 1 had the closest contact with the humans, sharing human 1’s bed. On May 7^th^ humans and cats were recruited to this case study. From human, nasal swabs, sputum, and fresh faecal samples were collected at intervals of 4–7 days. From cats, nasal swabs and fresh faecal samples were collected at intervals of 1–3 days until rtRT-PCR was negative for SARS-CoV-2. On May 7^th^ a nasal swab was collected from cat 1 and fresh faeces were collected from all 3 cats. Cat 1 tested positive for SARS-CoV-2 by rtRT-PCR in both nasal (Ct=31) and faecal sample (Ct=33), and cat 2 and 3 tested negative. In cats, at the last sampling, serum samples were also collected. Nasal samples were collected using flocked swabs; fresh faeces were stored in ziplock bags [15–17] and deposited in viral transport media.

The owners provided written consents at the time of the sample collection. For all the study, human sampling was performed by nurses and animal sampling was performed by veterinarians. Humans samples were tested at the Molecular Virology Laboratory, Pontificia Universidad Católica de Chile and animal samples were tested at the Animal Virology Laboratory, Universidad de Chile, La Pintana, Chile. The animal study was reviewed and approved by the Institutional Animal Care and Use Committee of Universidad de Chile, under protocol number 20370–VET–UCH, Biosafety Committee of Facultad de Ciencias Veterinarias y Pecuarias, Universidad de Chile under protocol number 161 and the human study was reviewed and approved under protocol 16–066 Scientific Ethical Committee on Health Sciences of Pontifical Universidad Católica de Chile.

### Real time RT–PCR

RNA from the samples was extracted using TRIzol™ Reagent (Invitrogen™, Carlsbad, CA, USA) following the instructions recommended by the manufacturer and eluted into 50 µL. The extracted RNA was subjected to rtRT-PCR to amplify a portion of ORF1b SARS-CoV-2 [18]. This PCR was previously validated at both laboratories independently, and is one of the RT-PCRs recommended by the WHO [19] to diagnose COVID-19. Positive and negative controls for PCR and extraction were included in each run.

### Serological testing

Serum samples were tested via a commercial ELISA test for SARS-CoV-2 antibody detection, the ID SCREEN® SARS-COV-2 Double Antigen Multi Species [20] (IDVET, Grabels, FRANCE).

### Viral isolation

Viral isolation was attempted in Vero E6 cells at Icahn School of Medicine at Mount Sinai, New York. Cells were cultured in minimum essential medium eagle 10% foetal bovine serum, 100 IU/mL penicillin, and 100 µg/mL streptomycin. Cells were seeded in 12-well culture plates and cultured at 37°C with 5% CO_2_ for 24-48 h. Cells were rinsed and inoculated with 300 µl of the direct sample and 1:10 and 1:100 dilutions. Mock-inoculated cells were used as negative controls. Cells were incubated at 37°C with 5% CO_2_ and monitored daily for cytopathic effect (CPE) for five days. Cell cultures with no CPE were frozen, thawed, and subjected to three blind passages with inoculation of fresh Vero E6 cell cultures with the lysates as described above.

### Viral sequencing

A selection of 25 rtRT-PCR positive specimens from humans and cats was submitted to the Icahn Institute for Data Science and Genomic Technology, Icahn School of Medicine at Mount Sinai, New York, NY for whole genome sequencing. Selection criteria were based on the cycle threshold values and inclusion of all cats and humans. The whole genome was amplified with a modified version of the Artic Consortium protocol [21] with custom tiling primers as previously described [22]. After amplification, viral sequencing was attempted on 12 positive samples, four human and eight from cat. Purified amplicons were used for library preparation with Nextera XT DNA Sample preparation Kit (Illumina, FC-131-1096) and sequenced on an Illumina MiSeq instrument (2 × 150 nt). SARS-CoV-2 genomes were assembled and annotated using a reference-based analysis pipeline as previously described [22].

### Sequence data analysis

Six SARS-CoV-2 genome sequences were analysed. These sequences were obtained from the clinical nasopharyngeal specimens of two humans that reside in the household: Human 1 (hCoV-19/Chile/Santiago/op31d5/2020; Human 1 05-07-20) and Human 2 (hCoV-19/Chile/Santiago/op32d10/2020); (Human 2 05-11-20), and four sequences from their cats – two of these were collected from nasal swabs of cat 2 on May 12^th^, 13^th^ and 16^th^, and a nasal sample from cat 3 collected on May 17^th^. Completeness of sequencing coverage and mutations were estimated by comparing the alignment with the reference sequence Wuhan-Hu-1 (GenBank accession NC_045512.2). Sequences were aligned to a set of reference SARS-CoV-2 sequences including all Chilean sequences submitted to GISAID [22] (n=167), sequences with highest similarity deposited on GenBank identified using BLAST [23] (n=337), and a total of 1000 sequences randomly selected from the GISAID repository using FastaUtils package in R [24]. The sequences were aligned using MAFFT v7.402 using CIPRESS computational resources [25]. The aligned sequences were visualized in AliView v1.26 [26]. Duplicate sequences were removed and non-coding regions, 5’ and 3’ UTR were trimmed. A phylogeny was estimated using the maximum likelihood method available in IQ-TREE v1.6.7 employing the GTR + F+I+Γ_4_ model of nucleotide substitution with 1000 bootstrap replications. The phylogenetic tree was visualized using Figtree. SARS-CoV-2 lineages were identified using the Pangolin COVID-19 Lineage Assigner web application [27].

## Results

### Naturally infected cats excreted SARS-CoV-2 RNA in a different pattern to that of humans

**Figure 1.**
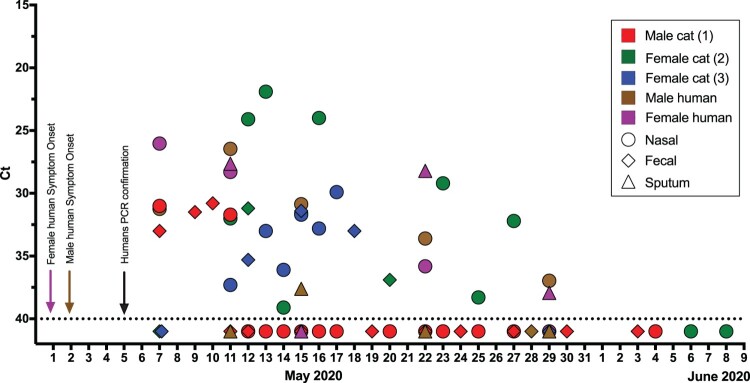
RNA detection of SARS-CoV-2. The horizontal axis represents the sampling date. Nasal swabs (circle), faecal samples (diamond), and sputum (triangle) samples are coloured per individual. Negative samples are illustrated as Ct 41 (below the dotted line). Arrows with the corresponding caption colours indicate the day of symptom onset and the date of the SARS-CoV-2 diagnosis of humans.

To determine the duration and dynamics of SARS-Cov-2 RNA detection in cat excretions under natural conditions, we assessed, by real time RT–PCR (rtRT-PCR), the presence of SARS-CoV-2 RNA in nasal and faecal samples from three companion cats over a 40-day time period. The cats acquired the SARS-CoV-2 infection naturally (rather than experimentally) and were residing in their usual household during the sampling period. Cat 2 had a pre-existing chronic rhinosinusitis as comorbidity. The household included two humans, and both were confirmed positive for SARS-CoV-2 before sampling of the cats commenced. Nasal, sputum, and faecal samples were taken from the humans and also assessed by rtRT-PCR for the presence of SARS-CoV-2 RNA over the time period in which the cats were sampled. All three cats had detectable viral RNA in both nasal swabs and faecal samples, with cycle threshold (Ct) values over 21.9 (Figure 1). In both humans, nasal and sputum samples showed Ct values ranging from 26 to 40. However, all human faecal samples showed negative rtRT-PCR results. The duration for which SARS-CoV-2 RNA could be detected in cat samples varied between the three cats sampled (5 days for cat 1, 8 days for cat 3, 17 for cat 2). Even at the extreme end of the range, the duration of detection was considerably shorter than the human samples. SARS-CoV-2 RNA was detected in the human samples up to 25 days after the first RNA detection. The dynamics of detectable viral RNA in the cat samples were more erratic compared to that for detectable viral RNA in humans from the same household. For example, a female cat (cat 2) presented an intermittent RNA detection for at least 17 days.

To assess the infection of the cats and humans, we made clinical observations for symptoms of SARS-CoV-2 infection and performed serological testing. Cat 1 presented with mild clinical signs including dullness, lethargy, and coughing without fever. Cats 2 and 3 were asymptomatic. On the last sampling day on June 9^th^, cat 2 tested positive for SARS-CoV-2 antibodies by ELISA. Both humans displayed COVID-19 symptoms including fever, lethargy, and coughing. However, the female human was more clinically affected and was the only human that tested positive for SARS-CoV-2 antibodies by ELISA starting at May 11, 2020.

Multiple attempts to isolate the virus from low Ct (high levels of SARS-CoV-2 RNA; 21.9 −24.1) samples were made without success.

### Human and cat origin SARS-CoV-2 genome sequences are identical

Of the specimens sent for genome sequencing one had coverage of 69·3% (human 2, day 10, nasopharyngeal) and five had a genome coverage >98% (three nasal swabs from cat 2, one nasal swab from cat 3, and one nasopharyngeal from human 1) (Appendix Figure). The remaining sequenced samples had a coverage <50% and were not included in the analyses. Sequences were deposited in GenBank (accession numbers Pending). Using the Pangolin tool [27] all sequences were assigned to lineage B.1.1, a major European lineage that was exported to the rest of the world from Europe. Of the 178 Chilean sequences included in this analysis (from GISAID and GenBank), 34 belong to this lineage.

**Figure 2.**
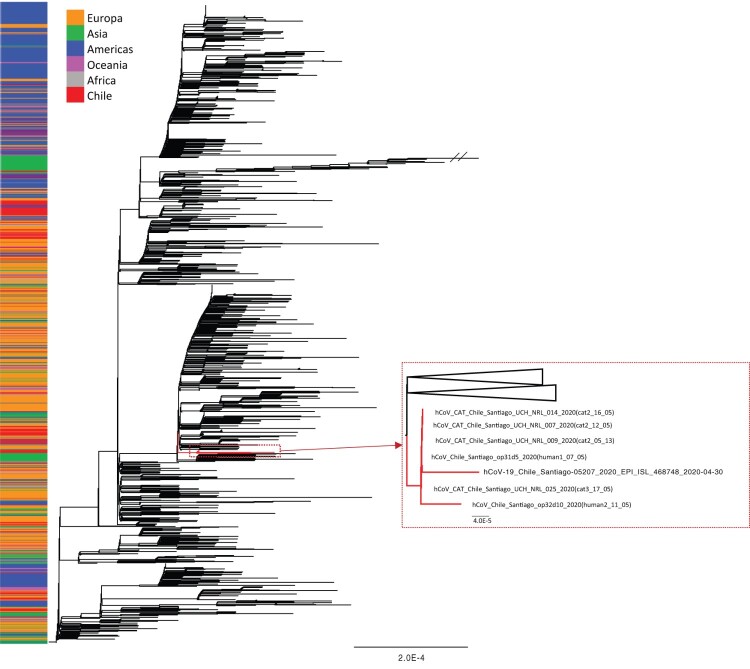
Phylogenetic tree estimated with the all available Chilean SARS-CoV-2 sequences and a random sample of available background sequences. Near to complete genome sequences of cat 2 (n=3), cat 3 (n=1), and both owners formed a monophyletic group. An additional human sequence collected in the same time period and area similarly fell into this cluster. The phylogeny was estimated using the maximum likelihood method available in IQ-TREE v1.6.7 employing the GTR + F+I+Γ4 model of nucleotide substitution with 1000 bootstrap replications.

We conducted a phylogenetic analysis to investigate any relationship between the SARS-CoV-2 RNA excreted by different cats and humans within the household. All six sequences obtained fell into a monophyletic group defined by a C25344 T (S1261F/Spike) nucleotide substitution (Figure 2). Notably, there were no nucleotide differences between the viral sequences obtained from cats and one of the household humans (hCoV-19/Chile/Santiago/op31d5/2020; Human 1 05-07-20). The viral sequence from the female human (hCoV-19/Chile/Santiago/op32d10/2020); (Human 2 05-11-20) contained two polymorphisms compared to the rest of the sequences (C11511 T/T3749I/nsp6) and (C13740 T/syn/RdRp) but fell in the same group. An epidemiologically unrelated Chilean SARS-CoV-2 sequence (GISAID accession number EPI_ISL_468748) collected in the same city on April 30^th^ was also grouped within this cluster of 6 sequences.

## Discussion

Several reports have confirmed the susceptibility of cats to SARS-CoV-2 infection in natural conditions and viral transmissibility between cats under experimental conditions [9,14,28,29]. However, these do not provide insight into the infection dynamics of cats in natural settings. This is the first report to describe the RNA excretion profiles of SARS-CoV-2 in naturally infected cats. The viral RNA in samples may indirectly denote SARS-CoV-2 viral excretion and can serve, with limitations, as a proxy for active viral replication. Indeed, SARS-CoV-2 antibodies detected by ELISA confirmed the infection in cat 2. Our household study thus provides preliminary guidance for managing the care and quarantine of cats in households in which SARS-CoV-2 is present.

The results, particularly the similarity of genome sequences depicted in the phylogenetic analysis, strongly support the idea that SARS-CoV-2 can be transmitted between humans and cats living in the same household. It is suspected that the humans were infected following exposure to SARS-CoV-2-positive neighbours around April 25th. We think it likely that cat 1 was the first infected of the three cats because this animal had closer contact than the other two cats with human 1 (male), sharing his bed. This is supported by our observation that viral RNA from human 1 and the cats had identical sequences whereas the female human’s viral RNA differed by two polymorphisms. Transmission between humans and cats in the same household accords with the literature on viral infections in animals. Several animal species are permissive for virus infection and replication in the upper respiratory tract [1] and it has been suggested that the species barrier of SARS-CoV-2 might be weak since the ACE2 host receptor may allow virus attachment even after some amino acid changes [30]. SARS-CoV-2 can be transmitted to animals by direct or indirect contact with contaminated surfaces or excretions [31]. For cats [1] and ferrets [32] respiratory transmission can be mediated by droplets or aerosols. Our findings of positive SARS-CoV-2 RNA detection in nasal samples (ranging from 5 to 17 days) and in faecal samples (ranging from 4 to 10 days) in all three cats, further support recommendations for avoiding close contact with companion animals when an infection is suspected in owners or family members.

Whether all cats in the study household were infected from humans, or whether some transmission occurred between animals, cannot be determined. Nevertheless, the possibility of some transmission between cats in the household is suggested by the 4-day delay in cats 2 and 3 testing positive for SARS-CoV-2 by rtRT-PCR compared to cat 1. All three cats had direct contact with each other. Transmission between cats in the household would be consistent with the findings of Shi et al. [1] who studied transmission between cats in an experimental setting. In that study, inoculated and infected sub-adult cats were placed next to susceptible cats in an adjacent cage (non-direct contact). Of the three replicates, one exposed cat gave rise to a positive rtRT-PCR on the 5th day post-exposure [8]. To date, however, we have only identified three rtRT-PCR-positive cats, with one of these also positive by ELISA, and one cat of another household similarly positive by ELISA. Consequently, it is likely that cat-to-cat transmission events are not as common as human-to-human transmission. The lack of virus isolation was most likely for storage and shipping difficulties. The samples were not always stored at −80°C, due to limitations during the quarantine. Also, the overseas shipping from Chile to the USA may affect the cold chain.

In summary, at present, there is insufficient data to identify and prioritize the drivers for the infection of companion animals [33]. Reported cases in animals correspond to independent and sporadic events. The findings presented here show that cats seemingly have different and shorter patterns of excretion for SARS-CoV-2 RNA compared to humans. These results highlight a need for large-scale epidemiological analysis of SARS-CoV-2 infection dynamics to support the establishment of preventive measures in real-life human-animal relationships.

## Supplementary Material

Suplementary_Information_1_fnal.xlsx

Supplementary_Information_2.pdf

## Data Availability

Sequences that support the results of this study have been deposited at GenBank with accession numbers (MW064259-MW064264). Animal testing results are detailed in supplementary information.
