# Supplementary material for: A household case evidences shorter shedding of SARS-CoV-2 in naturally infected cats compared to their human owners
Source: Emerg Microbes Infect. 2021 Mar 5;10(1):376–83. doi: 10.1080/22221751.2020.1863132 (PMC7939552; doi:10.1080/22221751.2020.1863132)
Supplement: Supplementary_Information_2.pdf [file TEMI_A_1863132_SM4811.pdf]

We gratefully acknowledge the following Authors from the Originating laboratories responsible for obtaining the specimens, as well as the Submitting laboratories where the genome data were generated and shared via GISAID, on which this research is based.

All Submitters of data may be contacted directly via www.gisaid.org

Accession ID	Originating Laboratory	Submitting Laboratory	Authors
EPI_ISL_414577, EPI_ISL_414578	Hospital de Talca, Chile	Instituto de Salud Publica de Chile	Andrés E. Castillo, Bárbara Parra, Paz Tapia, Alejandra Acevedo, Jaime Lagos, Winston Andrade, Loredana Arata, Gabriel Leal, Gisselle Barra, Carolina Tambley, Javier Tognarelli, Patricia Bustos, Soledad Ulloa, Rodrigo Fasce, Jorge Fernández.
EPI_ISL_414579	Clinica Alemana de Santiago, Chile	Instituto de Salud Publica de Chile	Andrés E. Castillo, Bárbara Parra, Paz Tapia, Alejandra Acevedo, Jaime Lagos, Winston Andrade, Loredana Arata, Gabriel Leal, Gisselle Barra, Carolina Tambley, Javier Tognarelli, Patricia Bustos, Soledad Ulloa, Rodrigo Fasce, Jorge Fernández.
EPI_ISL_414580	Clinica Santa Maria, Santiago, Chile	Instituto de Salud Publica de Chile	Andrés E. Castillo, Bárbara Parra, Paz Tapia, Alejandra Acevedo, Jaime Lagos, Winston Andrade, Loredana Arata, Gabriel Leal, Gisselle Barra, Carolina Tambley, Javier Tognarelli, Patricia Bustos, Soledad Ulloa, Rodrigo Fasce, Jorge Fernández.
EPI_ISL_415658, EPI_ISL_415660, EPI_ISL_415661	Laboratory of Molecular Virology, Pontificia Universidad Católica de Chile	MSHS Pathogen Surveillance Program	Rafael A. Medina, Pablo Vial, Tamara Garcia, Eileen Serrano, Ana Silvia Gonzalez-Reiche, Zenab Khan, Mitchell Sullivan, Ajay Obla, Matthew Hernandez, Hala Alshammary, Juan Soto, Shwetha Sridhar Hara, Ying-Chih Wang, Melissa Smith, Robert Sebra, Viviana Simon, Harm van Bakel
EPI_ISL_445245	CLINICA ALEMANA DE SANTIAGO S.A.	Instituto de Salud Publica de Chile	Andrés E Castillo, Bárbara Parra,Paz Tapia, Jaime Lagos, Loredana Arata, Alejandra Acevedo, Winston Andrade, Gabriel Leal, Carolina Tambley, Patricia Bustos, Rodrigo Fasce, Jorge Fernandez
EPI_ISL_445246	HOSPITAL PUERTO MONTT	Instituto de Salud Publica de Chile	Andrés E Castillo, Bárbara Parra,Paz Tapia, Jaime Lagos, Loredana Arata, Alejandra Acevedo, Winston Andrade, Gabriel Leal, Carolina Tambley, Patricia Bustos, Rodrigo Fasce, Jorge Fernandez
EPI_ISL_445247	UNIVERSIDAD DE LOS ANDES	Instituto de Salud Publica de Chile	Andrés E Castillo, Bárbara Parra,Paz Tapia, Jaime Lagos, Loredana Arata, Alejandra Acevedo, Winston Andrade, Gabriel Leal, Carolina Tambley, Patricia Bustos, Rodrigo Fasce, Jorge Fernandez
EPI_ISL_445248	CLINICA ALEMANA DE SANTIAGO S.A.	Instituto de Salud Publica de Chile	Andrés E Castillo, Bárbara Parra,Paz Tapia, Jaime Lagos, Loredana Arata, Alejandra Acevedo, Winston Andrade, Gabriel Leal, Carolina Tambley, Patricia Bustos, Rodrigo Fasce, Jorge Fernandez
EPI_ISL_445249	CLINICA SANTA MARIA S.A.	Instituto de Salud Publica de Chile	Andrés E Castillo, Bárbara Parra,Paz Tapia, Jaime Lagos, Loredana Arata, Alejandra Acevedo, Winston Andrade, Gabriel Leal, Carolina Tambley, Patricia Bustos, Rodrigo Fasce, Jorge Fernandez
EPI_ISL_445250	CLINICA ALEMANA DE SANTIAGO S.A.	Instituto de Salud Publica de Chile	Andrés E Castillo, Bárbara Parra,Paz Tapia, Jaime Lagos, Loredana Arata, Alejandra Acevedo, Winston Andrade, Gabriel Leal, Carolina Tambley, Patricia Bustos, Rodrigo Fasce, Jorge Fernandez
EPI_ISL_445251	HOSPITAL DE CARABINEROS	Instituto de Salud Publica de Chile	Andrés E Castillo, Bárbara Parra,Paz Tapia, Jaime Lagos, Loredana Arata, Alejandra Acevedo, Winston Andrade, Gabriel Leal, Carolina Tambley, Patricia Bustos, Rodrigo Fasce, Jorge Fernandez
EPI_ISL_445252	PONTIFICIA U. CATOLICA FAC. MEDICINA	Instituto de Salud Publica de Chile	Andrés E Castillo, Bárbara Parra,Paz Tapia, Jaime Lagos, Loredana Arata, Alejandra Acevedo, Winston Andrade, Gabriel Leal, Carolina Tambley, Patricia Bustos, Rodrigo Fasce, Jorge Fernandez
EPI_ISL_445253, EPI_ISL_445254, EPI_ISL_445255	CLINICA ALEMANA DE SANTIAGO S.A.	Instituto de Salud Publica de Chile	Andrés E Castillo, Bárbara Parra,Paz Tapia, Jaime Lagos, Loredana Arata, Alejandra Acevedo, Winston Andrade, Gabriel Leal, Carolina Tambley, Patricia Bustos, Rodrigo Fasce, Jorge Fernandez
EPI_ISL_445256	CLINICA LAS CONDES S.A.	Instituto de Salud Publica de Chile	Andrés E Castillo, Bárbara Parra,Paz Tapia, Jaime Lagos, Loredana Arata, Alejandra Acevedo, Winston Andrade, Gabriel Leal, Carolina Tambley, Patricia Bustos, Rodrigo Fasce, Jorge Fernandez
EPI_ISL_445257	CLINICA TABANCURA	Instituto de Salud Publica de Chile	Andrés E Castillo, Bárbara Parra,Paz Tapia, Jaime Lagos, Loredana Arata, Alejandra Acevedo, Winston Andrade, Gabriel Leal, Carolina Tambley, Patricia Bustos, Rodrigo Fasce, Jorge Fernandez
EPI_ISL_445258	CLINICA ALEMANA DE SANTIAGO S.A.	Instituto de Salud Publica de Chile	Andrés E Castillo, Bárbara Parra,Paz Tapia, Jaime Lagos, Loredana Arata, Alejandra Acevedo, Winston Andrade, Gabriel Leal, Carolina Tambley, Patricia Bustos, Rodrigo Fasce, Jorge Fernandez
EPI_ISL_445259	CLINICA LAS CONDES S.A.	Instituto de Salud Publica de Chile	Andrés E Castillo, Bárbara Parra,Paz Tapia, Jaime Lagos, Loredana Arata, Alejandra Acevedo, Winston Andrade, Gabriel Leal, Carolina Tambley, Patricia Bustos, Rodrigo Fasce, Jorge Fernandez
EPI_ISL_445260	CLINICA ALEMANA DE SANTIAGO S.A.	Instituto de Salud Publica de Chile	Andrés E Castillo, Bárbara Parra,Paz Tapia, Jaime Lagos, Loredana Arata, Alejandra Acevedo, Winston Andrade, Gabriel Leal, Carolina Tambley, Patricia Bustos, Rodrigo Fasce, Jorge Fernandez
EPI_ISL_445261	INTEGRAMEDICA LAB. CLINICO LTDA.	Instituto de Salud Publica de Chile	Andrés E Castillo, Bárbara Parra,Paz Tapia, Jaime Lagos, Loredana Arata, Alejandra Acevedo, Winston Andrade, Gabriel Leal, Carolina Tambley, Patricia Bustos, Rodrigo Fasce, Jorge Fernandez
EPI_ISL_445262	CLINICA TABANCURA	Instituto de Salud Publica de Chile	Andrés E Castillo, Bárbara Parra,Paz Tapia, Jaime Lagos, Loredana Arata, Alejandra Acevedo, Winston Andrade, Gabriel Leal, Carolina Tambley, Patricia Bustos, Rodrigo Fasce, Jorge Fernandez
EPI_ISL_445263	MEGASALUD SPA.	Instituto de Salud Publica de Chile	Andrés E Castillo, Bárbara Parra,Paz Tapia, Jaime Lagos, Loredana Arata, Alejandra Acevedo, Winston Andrade, Gabriel Leal, Carolina Tambley, Patricia Bustos, Rodrigo Fasce, Jorge Fernandez
EPI_ISL_445264	CLINICA REDSALUD VITACURA.	Instituto de Salud Publica de Chile	Andrés E Castillo, Bárbara Parra,Paz Tapia, Jaime Lagos, Loredana Arata, Alejandra Acevedo, Winston Andrade, Gabriel Leal, Carolina Tambley, Patricia Bustos, Rodrigo Fasce, Jorge Fernandez
EPI_ISL_445265	PONTIFICIA UNIVERSIDAD CATOLICA DE CHILE	Instituto de Salud Publica de Chile	Andrés E Castillo, Bárbara Parra,Paz Tapia, Jaime Lagos, Loredana Arata, Alejandra Acevedo, Winston Andrade, Gabriel Leal, Carolina Tambley, Patricia Bustos, Rodrigo Fasce, Jorge Fernandez
EPI_ISL_445266, EPI_ISL_445267	CENTRO ONCOLOGICO DEL NORTE	Instituto de Salud Publica de Chile	Andrés E Castillo, Bárbara Parra,Paz Tapia, Jaime Lagos, Loredana Arata, Alejandra Acevedo, Winston Andrade, Gabriel Leal, Carolina Tambley, Patricia Bustos, Rodrigo Fasce, Jorge Fernandez
EPI_ISL_445268, EPI_ISL_445269	HOSPITAL REG.LAUTARO NAVARRO AVARIA	Instituto de Salud Publica de Chile	Andrés E Castillo, Bárbara Parra,Paz Tapia, Jaime Lagos, Loredana Arata, Alejandra Acevedo, Winston Andrade, Gabriel Leal, Carolina Tambley, Patricia Bustos, Rodrigo Fasce, Jorge Fernandez
EPI_ISL_445270	HOSPITAL DR.HERNAN HENRIQUEZ ARAVENA	Instituto de Salud Publica de Chile	Andrés E Castillo, Bárbara Parra,Paz Tapia, Jaime Lagos, Loredana Arata, Alejandra Acevedo, Winston Andrade, Gabriel Leal, Carolina Tambley, Patricia Bustos, Rodrigo Fasce, Jorge Fernandez
EPI_ISL_445271	LABORATORIO CLINICA CHILLAN	Instituto de Salud Publica de Chile	Andrés E Castillo, Bárbara Parra,Paz Tapia, Jaime Lagos, Loredana Arata, Alejandra Acevedo, Winston Andrade, Gabriel Leal, Carolina Tambley, Patricia Bustos, Rodrigo Fasce, Jorge Fernandez
EPI_ISL_445272	CLINICA CIUDAD DEL MAR	Instituto de Salud Publica de Chile	Andrés E Castillo, Bárbara Parra,Paz Tapia, Jaime Lagos, Loredana Arata, Alejandra Acevedo, Winston Andrade, Gabriel Leal, Carolina Tambley, Patricia Bustos, Rodrigo Fasce, Jorge Fernandez
EPI_ISL_445273, EPI_ISL_445274	LABORATORIO TORRE MEDICA LTDA.	Instituto de Salud Publica de Chile	Andrés E Castillo, Bárbara Parra,Paz Tapia, Jaime Lagos, Loredana Arata, Alejandra Acevedo, Winston Andrade, Gabriel Leal, Carolina Tambley, Patricia Bustos, Rodrigo Fasce, Jorge Fernandez
EPI_ISL_445275, EPI_ISL_445276	HOSPITAL CLINICO FUSAT	Instituto de Salud Publica de Chile	Andrés E Castillo, Bárbara Parra,Paz Tapia, Jaime Lagos, Loredana Arata, Alejandra Acevedo, Winston Andrade, Gabriel Leal, Carolina Tambley, Patricia Bustos, Rodrigo Fasce, Jorge Fernandez
EPI_ISL_445277	FUNDACION DE SALUD EL TENIENTE	Instituto de Salud Publica de Chile	Andrés E Castillo, Bárbara Parra,Paz Tapia, Jaime Lagos, Loredana Arata, Alejandra Acevedo, Winston Andrade, Gabriel Leal, Carolina Tambley, Patricia Bustos, Rodrigo Fasce, Jorge Fernandez
EPI_ISL_445278	LABORATORIO TORRE MEDICA LTDA.	Instituto de Salud Publica de Chile	Andrés E Castillo, Bárbara Parra,Paz Tapia, Jaime Lagos, Loredana Arata, Alejandra Acevedo, Winston Andrade, Gabriel Leal, Carolina Tambley, Patricia Bustos, Rodrigo Fasce, Jorge Fernandez
EPI_ISL_445279	LABORATORIO INMUNOLAB SPA	Instituto de Salud Publica de Chile	Andrés E Castillo, Bárbara Parra,Paz Tapia, Jaime Lagos, Loredana Arata, Alejandra Acevedo, Winston Andrade, Gabriel Leal, Carolina Tambley, Patricia Bustos, Rodrigo Fasce, Jorge Fernandez
EPI_ISL_445280	HOSPITAL REG.LAUTARO NAVARRO AVARIA	Instituto de Salud Publica de Chile	Andrés E Castillo, Bárbara Parra,Paz Tapia, Jaime Lagos, Loredana Arata, Alejandra Acevedo, Winston Andrade, Gabriel Leal, Carolina Tambley, Patricia Bustos, Rodrigo Fasce, Jorge Fernandez
EPI_ISL_445281	HOSPITAL CLINICO DEL SUR	Instituto de Salud Publica de Chile	Andrés E Castillo, Bárbara Parra,Paz Tapia, Jaime Lagos, Loredana Arata, Alejandra Acevedo, Winston Andrade, Gabriel Leal, Carolina Tambley, Patricia Bustos, Rodrigo Fasce, Jorge Fernandez
EPI_ISL_445282, EPI_ISL_445283	CLINICA MAGALLANES S.A.	Instituto de Salud Publica de Chile	Andrés E Castillo, Bárbara Parra,Paz Tapia, Jaime Lagos, Loredana Arata, Alejandra Acevedo, Winston Andrade, Gabriel Leal, Carolina Tambley, Patricia Bustos, Rodrigo Fasce, Jorge Fernandez
EPI_ISL_445284	HOSPITAL REG.LAUTARO NAVARRO AVARIA	Instituto de Salud Publica de Chile	Andrés E Castillo, Bárbara Parra,Paz Tapia, Jaime Lagos, Loredana Arata, Alejandra Acevedo, Winston Andrade, Gabriel Leal, Carolina Tambley, Patricia Bustos, Rodrigo Fasce, Jorge Fernandez
EPI_ISL_445285	CLINICA CIUDAD DEL MAR	Instituto de Salud Publica de Chile	Andrés E Castillo, Bárbara Parra,Paz Tapia, Jaime Lagos, Loredana Arata, Alejandra Acevedo, Winston Andrade, Gabriel Leal, Carolina Tambley, Patricia Bustos, Rodrigo Fasce, Jorge Fernandez
EPI_ISL_445286	HOSPITAL HANGA ROA	Instituto de Salud Publica de Chile	Andrés E Castillo, Bárbara Parra,Paz Tapia, Jaime Lagos, Loredana Arata, Alejandra Acevedo, Winston Andrade, Gabriel Leal, Carolina Tambley, Patricia Bustos, Rodrigo Fasce, Jorge Fernandez
EPI_ISL_445287	CLINICA CIUDAD DEL MAR	Instituto de Salud Publica de Chile	Andrés E Castillo, Bárbara Parra,Paz Tapia, Jaime Lagos, Loredana Arata, Alejandra Acevedo, Winston Andrade, Gabriel Leal, Carolina Tambley, Patricia Bustos, Rodrigo Fasce, Jorge

			Fernandez
EPI_ISL_445288	HOSPITAL REG.LAUTARO NAVARRO AVARIA	Instituto de Salud Publica de Chile	Andrés E Castillo, Bárbara Parra,Paz Tapia, Jaime Lagos, Loredana Arata, Alejandra Acevedo, Winston Andrade, Gabriel Leal, Carolina Tambley, Patricia Bustos, Rodrigo Fasce, Jorge Fernandez
EPI_ISL_445289	HOSPITAL NAVAL PUERTO WILLIAMS	Instituto de Salud Publica de Chile	Andrés E Castillo, Bárbara Parra,Paz Tapia, Jaime Lagos, Loredana Arata, Alejandra Acevedo, Winston Andrade, Gabriel Leal, Carolina Tambley, Patricia Bustos, Rodrigo Fasce, Jorge Fernandez
EPI_ISL_445290, EPI_ISL_445291, EPI_ISL_445292	CLINICA MAGALLANES S.A.	Instituto de Salud Publica de Chile	Andrés E Castillo, Bárbara Parra,Paz Tapia, Jaime Lagos, Loredana Arata, Alejandra Acevedo, Winston Andrade, Gabriel Leal, Carolina Tambley, Patricia Bustos, Rodrigo Fasce, Jorge Fernandez
EPI_ISL_445293, EPI_ISL_445294, EPI_ISL_445295	HOSPITAL REG.LAUTARO NAVARRO AVARIA	Instituto de Salud Publica de Chile	Andrés E Castillo, Bárbara Parra,Paz Tapia, Jaime Lagos, Loredana Arata, Alejandra Acevedo, Winston Andrade, Gabriel Leal, Carolina Tambley, Patricia Bustos, Rodrigo Fasce, Jorge Fernandez
EPI_ISL_445296	HOSPITAL REGIONAL DE COYHAIQUE	Instituto de Salud Publica de Chile	Andrés E Castillo, Bárbara Parra,Paz Tapia, Jaime Lagos, Loredana Arata, Alejandra Acevedo, Winston Andrade, Gabriel Leal, Carolina Tambley, Patricia Bustos, Rodrigo Fasce, Jorge Fernandez
EPI_ISL_445297	CLINICA INTEGRAL S.A.	Instituto de Salud Publica de Chile	Andrés E Castillo, Bárbara Parra,Paz Tapia, Jaime Lagos, Loredana Arata, Alejandra Acevedo, Winston Andrade, Gabriel Leal, Carolina Tambley, Patricia Bustos, Rodrigo Fasce, Jorge Fernandez
EPI_ISL_445298	HOSPITAL DE SAN FERNANDO	Instituto de Salud Publica de Chile	Andrés E Castillo, Bárbara Parra,Paz Tapia, Jaime Lagos, Loredana Arata, Alejandra Acevedo, Winston Andrade, Gabriel Leal, Carolina Tambley, Patricia Bustos, Rodrigo Fasce, Jorge Fernandez
EPI_ISL_445299	CLINICA MAGALLANES S.A.	Instituto de Salud Publica de Chile	Andrés E Castillo, Bárbara Parra,Paz Tapia, Jaime Lagos, Loredana Arata, Alejandra Acevedo, Winston Andrade, Gabriel Leal, Carolina Tambley, Patricia Bustos, Rodrigo Fasce, Jorge Fernandez
EPI_ISL_445300	HOSPITAL DE RANCAGUA	Instituto de Salud Publica de Chile	Andrés E Castillo, Bárbara Parra,Paz Tapia, Jaime Lagos, Loredana Arata, Alejandra Acevedo, Winston Andrade, Gabriel Leal, Carolina Tambley, Patricia Bustos, Rodrigo Fasce, Jorge Fernandez
EPI_ISL_445301	HOSPITAL NAVAL PUERTO WILLIAMS	Instituto de Salud Publica de Chile	Andrés E Castillo, Bárbara Parra,Paz Tapia, Jaime Lagos, Loredana Arata, Alejandra Acevedo, Winston Andrade, Gabriel Leal, Carolina Tambley, Patricia Bustos, Rodrigo Fasce, Jorge Fernandez
EPI_ISL_445302	INSTITUTO MEDICO LEGAL	Instituto de Salud Publica de Chile	Andrés E Castillo, Bárbara Parra,Paz Tapia, Jaime Lagos, Loredana Arata, Alejandra Acevedo, Winston Andrade, Gabriel Leal, Carolina Tambley, Patricia Bustos, Rodrigo Fasce, Jorge Fernandez
EPI_ISL_445303	CTRO.DE SALUD FAMILIAR DR. RAUL YAZIGI	Instituto de Salud Publica de Chile	Andrés E Castillo, Bárbara Parra,Paz Tapia, Jaime Lagos, Loredana Arata, Alejandra Acevedo, Winston Andrade, Gabriel Leal, Carolina Tambley, Patricia Bustos, Rodrigo Fasce, Jorge Fernandez
EPI_ISL_445304	HOSPITAL SAN JUAN DE DIOS	Instituto de Salud Publica de Chile	Andrés E Castillo, Bárbara Parra,Paz Tapia, Jaime Lagos, Loredana Arata, Alejandra Acevedo, Winston Andrade, Gabriel Leal, Carolina Tambley, Patricia Bustos, Rodrigo Fasce, Jorge Fernandez
EPI_ISL_445305	HOSPITAL DE CARABINEROS	Instituto de Salud Publica de Chile	Andrés E Castillo, Bárbara Parra,Paz Tapia, Jaime Lagos, Loredana Arata, Alejandra Acevedo, Winston Andrade, Gabriel Leal, Carolina Tambley, Patricia Bustos, Rodrigo Fasce, Jorge Fernandez
EPI_ISL_445306	CLINICA UC SAN CARLOS DE APOQUINDO	Instituto de Salud Publica de Chile	Andrés E Castillo, Bárbara Parra,Paz Tapia, Jaime Lagos, Loredana Arata, Alejandra Acevedo, Winston Andrade, Gabriel Leal, Carolina Tambley, Patricia Bustos, Rodrigo Fasce, Jorge Fernandez
EPI_ISL_445307	HOSPITAL SAN JOSE DE MAIPO	Instituto de Salud Publica de Chile	Andrés E Castillo, Bárbara Parra,Paz Tapia, Jaime Lagos, Loredana Arata, Alejandra Acevedo, Winston Andrade, Gabriel Leal, Carolina Tambley, Patricia Bustos, Rodrigo Fasce, Jorge Fernandez
EPI_ISL_445308	HOSPITAL EL CARMEN DR.LUIS VALENTIN F.	Instituto de Salud Publica de Chile	Andrés E Castillo, Bárbara Parra,Paz Tapia, Jaime Lagos, Loredana Arata, Alejandra Acevedo, Winston Andrade, Gabriel Leal, Carolina Tambley, Patricia Bustos, Rodrigo Fasce, Jorge Fernandez
EPI_ISL_445309	MUTUAL DE SEGURIDAD C.CH.C.	Instituto de Salud Publica de Chile	Andrés E Castillo, Bárbara Parra,Paz Tapia, Jaime Lagos, Loredana Arata, Alejandra Acevedo, Winston Andrade, Gabriel Leal, Carolina Tambley, Patricia Bustos, Rodrigo Fasce, Jorge Fernandez
EPI_ISL_445310	HOSPITAL DR.SOTERO DEL RIO	Instituto de Salud Publica de Chile	Andrés E Castillo, Bárbara Parra,Paz Tapia, Jaime Lagos, Loredana Arata, Alejandra Acevedo, Winston Andrade, Gabriel Leal, Carolina Tambley, Patricia Bustos, Rodrigo Fasce, Jorge Fernandez
EPI_ISL_445311	MEGASALUD S.A.	Instituto de Salud Publica de Chile	Andrés E Castillo, Bárbara Parra,Paz Tapia, Jaime Lagos, Loredana Arata, Alejandra Acevedo, Winston Andrade, Gabriel Leal, Carolina Tambley, Patricia Bustos, Rodrigo Fasce, Jorge Fernandez
EPI_ISL_445312	CLINICA UC SAN CARLOS DE APOQUINDO	Instituto de Salud Publica de Chile	Andrés E Castillo, Bárbara Parra,Paz Tapia, Jaime Lagos, Loredana Arata, Alejandra Acevedo, Winston Andrade, Gabriel Leal, Carolina Tambley, Patricia Bustos, Rodrigo Fasce, Jorge Fernandez
EPI_ISL_445313, EPI_ISL_445314	HOSPITAL EL CARMEN DR.LUIS VALENTIN F.	Instituto de Salud Publica de Chile	Andrés E Castillo, Bárbara Parra,Paz Tapia, Jaime Lagos, Loredana Arata, Alejandra Acevedo, Winston Andrade, Gabriel Leal, Carolina Tambley, Patricia Bustos, Rodrigo Fasce, Jorge Fernandez
EPI_ISL_445315	CLINICA UC SAN CARLOS DE APOQUINDO	Instituto de Salud Publica de Chile	Andrés E Castillo, Bárbara Parra,Paz Tapia, Jaime Lagos, Loredana Arata, Alejandra Acevedo, Winston Andrade, Gabriel Leal, Carolina Tambley, Patricia Bustos, Rodrigo Fasce, Jorge Fernandez
EPI_ISL_445316	CESFAM BALMACEDA DE RENCA	Instituto de Salud Publica de Chile	Andrés E Castillo, Bárbara Parra,Paz Tapia, Jaime Lagos, Loredana Arata, Alejandra Acevedo, Winston Andrade, Gabriel Leal, Carolina Tambley, Patricia Bustos, Rodrigo Fasce, Jorge Fernandez
EPI_ISL_445317	UNIV.DE CHILE HOSP.CLINICO	Instituto de Salud Publica de Chile	Andrés E Castillo, Bárbara Parra,Paz Tapia, Jaime Lagos, Loredana Arata, Alejandra Acevedo, Winston Andrade, Gabriel Leal, Carolina Tambley, Patricia Bustos, Rodrigo Fasce, Jorge Fernandez
EPI_ISL_445318	C.DE SALUD FAMILIAR PABLO NERUDA	Instituto de Salud Publica de Chile	Andrés E Castillo, Bárbara Parra,Paz Tapia, Jaime Lagos, Loredana Arata, Alejandra Acevedo, Winston Andrade, Gabriel Leal, Carolina Tambley, Patricia Bustos, Rodrigo Fasce, Jorge Fernandez
EPI_ISL_445319	HOSPITAL FELIX BULNES	Instituto de Salud Publica de Chile	Andrés E Castillo, Bárbara Parra,Paz Tapia, Jaime Lagos, Loredana Arata, Alejandra Acevedo, Winston Andrade, Gabriel Leal, Carolina Tambley, Patricia Bustos, Rodrigo Fasce, Jorge Fernandez
EPI_ISL_445320	C.C.SALUD FAMILIAR PADRE FELIX DONOSO G.	Instituto de Salud Publica de Chile	Andrés E Castillo, Bárbara Parra,Paz Tapia, Jaime Lagos, Loredana Arata, Alejandra Acevedo, Winston Andrade, Gabriel Leal, Carolina Tambley, Patricia Bustos, Rodrigo Fasce, Jorge Fernandez
EPI_ISL_445321	HOSPITAL FELIX BULNES	Instituto de Salud Publica de Chile	Andrés E Castillo, Bárbara Parra,Paz Tapia, Jaime Lagos, Loredana Arata, Alejandra Acevedo, Winston Andrade, Gabriel Leal, Carolina Tambley, Patricia Bustos, Rodrigo Fasce, Jorge Fernandez
EPI_ISL_445322	UNIV.DE CHILE HOSP.CLINICO	Instituto de Salud Publica de Chile	Andrés E Castillo, Bárbara Parra,Paz Tapia, Jaime Lagos, Loredana Arata, Alejandra Acevedo, Winston Andrade, Gabriel Leal, Carolina Tambley, Patricia Bustos, Rodrigo Fasce, Jorge Fernandez
EPI_ISL_445323	HOSP.ENFERMEDADES INFECCIOSAS	Instituto de Salud Publica de Chile	Andrés E Castillo, Bárbara Parra,Paz Tapia, Jaime Lagos, Loredana Arata, Alejandra Acevedo, Winston Andrade, Gabriel Leal, Carolina Tambley, Patricia Bustos, Rodrigo Fasce, Jorge Fernandez
EPI_ISL_445324	INTEGRAMEDICA S.A	Instituto de Salud Publica de Chile	Andrés E Castillo, Bárbara Parra,Paz Tapia, Jaime Lagos, Loredana Arata, Alejandra Acevedo, Winston Andrade, Gabriel Leal, Carolina Tambley, Patricia Bustos, Rodrigo Fasce, Jorge Fernandez
EPI_ISL_445325	HOSPITAL DR.SOTERO DEL RIO	Instituto de Salud Publica de Chile	Andrés E Castillo, Bárbara Parra,Paz Tapia, Jaime Lagos, Loredana Arata, Alejandra Acevedo, Winston Andrade, Gabriel Leal, Carolina Tambley, Patricia Bustos, Rodrigo Fasce, Jorge Fernandez
EPI_ISL_445326	ASISTENCIA PUBLICA DR.ALEJANDRO DEL RIO	Instituto de Salud Publica de Chile	Andrés E Castillo, Bárbara Parra,Paz Tapia, Jaime Lagos, Loredana Arata, Alejandra Acevedo, Winston Andrade, Gabriel Leal, Carolina Tambley, Patricia Bustos, Rodrigo Fasce, Jorge Fernandez
EPI_ISL_445327	HOSPITAL PADRE HURTADO	Instituto de Salud Publica de Chile	Andrés E Castillo, Bárbara Parra,Paz Tapia, Jaime Lagos, Loredana Arata, Alejandra Acevedo, Winston Andrade, Gabriel Leal, Carolina Tambley, Patricia Bustos, Rodrigo Fasce, Jorge Fernandez
EPI_ISL_445328	MEGASALUD S.A.	Instituto de Salud Publica de Chile	Andrés E Castillo, Bárbara Parra,Paz Tapia, Jaime Lagos, Loredana Arata, Alejandra Acevedo, Winston Andrade, Gabriel Leal, Carolina Tambley, Patricia Bustos, Rodrigo Fasce, Jorge Fernandez
EPI_ISL_445329	HOSPITAL DR.SOTERO DEL RIO	Instituto de Salud Publica de Chile	Andrés E Castillo, Bárbara Parra,Paz Tapia, Jaime Lagos, Loredana Arata, Alejandra Acevedo, Winston Andrade, Gabriel Leal, Carolina Tambley, Patricia Bustos, Rodrigo Fasce, Jorge Fernandez
EPI_ISL_445330	CLINICA VESPUCIO S. A.	Instituto de Salud Publica de Chile	Andrés E Castillo, Bárbara Parra,Paz Tapia, Jaime Lagos, Loredana Arata, Alejandra Acevedo, Winston Andrade, Gabriel Leal, Carolina Tambley, Patricia Bustos, Rodrigo Fasce, Jorge Fernandez
EPI_ISL_445331, EPI_ISL_445332	HOSPITAL HERMINDA MARTIN CHILLAN	Instituto de Salud Publica de Chile	Andrés E Castillo, Bárbara Parra,Paz Tapia, Jaime Lagos, Loredana Arata, Alejandra Acevedo, Winston Andrade, Gabriel Leal, Carolina Tambley, Patricia Bustos, Rodrigo Fasce, Jorge Fernandez
EPI_ISL_445333	LABORATORIO CLINICA UNIVERSITARIA DE CONCEPCION	Instituto de Salud Publica de Chile	Andrés E Castillo, Bárbara Parra,Paz Tapia, Jaime Lagos, Loredana Arata, Alejandra Acevedo, Winston Andrade, Gabriel Leal, Carolina Tambley, Patricia Bustos, Rodrigo Fasce, Jorge Fernandez
EPI_ISL_445334	CLINICA UNIVERSITARIA DE PUERTO MONTT S.A.	Instituto de Salud Publica de Chile	Andrés E Castillo, Bárbara Parra,Paz Tapia, Jaime Lagos, Loredana Arata, Alejandra Acevedo, Winston Andrade, Gabriel Leal, Carolina Tambley, Patricia Bustos, Rodrigo Fasce, Jorge Fernandez
EPI_ISL_445335	HOSPITAL DE CALBUCO	Instituto de Salud Publica de Chile	Andrés E Castillo, Bárbara Parra,Paz Tapia, Jaime Lagos, Loredana Arata, Alejandra Acevedo, Winston Andrade, Gabriel Leal, Carolina Tambley, Patricia Bustos, Rodrigo Fasce, Jorge Fernandez
EPI_ISL_445336	HOSPITAL LAS HIGUERAS DE TALCAHUANO	Instituto de Salud Publica de Chile	Andrés E Castillo, Bárbara Parra,Paz Tapia, Jaime Lagos, Loredana Arata, Alejandra Acevedo, Winston Andrade, Gabriel Leal, Carolina Tambley, Patricia Bustos, Rodrigo Fasce, Jorge Fernandez
EPI_ISL_445337	HOSPITAL HANGA ROA	Instituto de Salud Publica de Chile	Andrés E Castillo, Bárbara Parra,Paz Tapia, Jaime Lagos, Loredana Arata, Alejandra Acevedo, Winston Andrade, Gabriel Leal, Carolina Tambley, Patricia Bustos, Rodrigo Fasce, Jorge Fernandez
EPI_ISL_445338, EPI_ISL_445339, EPI_ISL_445340, EPI_ISL_445341, EPI_ISL_445342, EPI_ISL_445343, EPI_ISL_445344, EPI_ISL_445345, EPI_ISL_445346, EPI_ISL_445347, EPI_ISL_445348			

see above	HOSPITAL DR.HERNAN HENRIQUEZ ARAVENA	Instituto de Salud Publica de Chile	Andrés E Castillo, Bárbara Parra,Paz Tapia, Jaime Lagos, Loredana Arata, Alejandra Acevedo, Winston Andrade, Gabriel Leal, Carolina Tambley, Patricia Bustos, Rodrigo Fasce, Jorge Fernandez
EPI_ISL_445349, EPI_ISL_445350, EPI_ISL_445351	HOSPITAL SAN JUAN DE DIOS	Instituto de Salud Publica de Chile	Andrés E Castillo, Bárbara Parra,Paz Tapia, Jaime Lagos, Loredana Arata, Alejandra Acevedo, Winston Andrade, Gabriel Leal, Carolina Tambley, Patricia Bustos, Rodrigo Fasce, Jorge Fernandez
EPI_ISL_445352	HOSPITAL DEL PROFESOR	Instituto de Salud Publica de Chile	Andrés E Castillo, Bárbara Parra,Paz Tapia, Jaime Lagos, Loredana Arata, Alejandra Acevedo, Winston Andrade, Gabriel Leal, Carolina Tambley, Patricia Bustos, Rodrigo Fasce, Jorge Fernandez
EPI_ISL_445353	HOSPITAL PADRE HURTADO	Instituto de Salud Publica de Chile	Andrés E Castillo, Bárbara Parra,Paz Tapia, Jaime Lagos, Loredana Arata, Alejandra Acevedo, Winston Andrade, Gabriel Leal, Carolina Tambley, Patricia Bustos, Rodrigo Fasce, Jorge Fernandez
EPI_ISL_445354	HOSPITAL DE CARABINEROS	Instituto de Salud Publica de Chile	Andrés E Castillo, Bárbara Parra,Paz Tapia, Jaime Lagos, Loredana Arata, Alejandra Acevedo, Winston Andrade, Gabriel Leal, Carolina Tambley, Patricia Bustos, Rodrigo Fasce, Jorge Fernandez
EPI_ISL_445355	MUTUAL DE SEGURIDAD C.CH.C.	Instituto de Salud Publica de Chile	Andrés E Castillo, Bárbara Parra,Paz Tapia, Jaime Lagos, Loredana Arata, Alejandra Acevedo, Winston Andrade, Gabriel Leal, Carolina Tambley, Patricia Bustos, Rodrigo Fasce, Jorge Fernandez
EPI_ISL_445356	PONTIFICIA U. CATOLICA SERV. LABORATORIO	Instituto de Salud Publica de Chile	Andrés E Castillo, Bárbara Parra,Paz Tapia, Jaime Lagos, Loredana Arata, Alejandra Acevedo, Winston Andrade, Gabriel Leal, Carolina Tambley, Patricia Bustos, Rodrigo Fasce, Jorge Fernandez
EPI_ISL_445357	INTEGRAMEDICA CENTROS MEDICOS S.A.	Instituto de Salud Publica de Chile	Andrés E Castillo, Bárbara Parra,Paz Tapia, Jaime Lagos, Loredana Arata, Alejandra Acevedo, Winston Andrade, Gabriel Leal, Carolina Tambley, Patricia Bustos, Rodrigo Fasce, Jorge Fernandez
EPI_ISL_445358	MEGASALUD SPA.	Instituto de Salud Publica de Chile	Andrés E Castillo, Bárbara Parra,Paz Tapia, Jaime Lagos, Loredana Arata, Alejandra Acevedo, Winston Andrade, Gabriel Leal, Carolina Tambley, Patricia Bustos, Rodrigo Fasce, Jorge Fernandez
EPI_ISL_445359	HOSP. SANTIAGO ORIENTE DR. LUIS TISNE B.	Instituto de Salud Publica de Chile	Andrés E Castillo, Bárbara Parra,Paz Tapia, Jaime Lagos, Loredana Arata, Alejandra Acevedo, Winston Andrade, Gabriel Leal, Carolina Tambley, Patricia Bustos, Rodrigo Fasce, Jorge Fernandez
EPI_ISL_445360	HOSPITAL DEL PROFESOR	Instituto de Salud Publica de Chile	Andrés E Castillo, Bárbara Parra,Paz Tapia, Jaime Lagos, Loredana Arata, Alejandra Acevedo, Winston Andrade, Gabriel Leal, Carolina Tambley, Patricia Bustos, Rodrigo Fasce, Jorge Fernandez
EPI_ISL_445361	CLINICA UC SAN CARLOS DE APOQUINDO	Instituto de Salud Publica de Chile	Andrés E Castillo, Bárbara Parra,Paz Tapia, Jaime Lagos, Loredana Arata, Alejandra Acevedo, Winston Andrade, Gabriel Leal, Carolina Tambley, Patricia Bustos, Rodrigo Fasce, Jorge Fernandez
EPI_ISL_445362	BUPA SERVICIOS CLINICOS S.A	Instituto de Salud Publica de Chile	Andrés E Castillo, Bárbara Parra,Paz Tapia, Jaime Lagos, Loredana Arata, Alejandra Acevedo, Winston Andrade, Gabriel Leal, Carolina Tambley, Patricia Bustos, Rodrigo Fasce, Jorge Fernandez
EPI_ISL_445363	ASISTENCIA PUBLICA DR.ALEJANDRO DEL RIO	Instituto de Salud Publica de Chile	Andrés E Castillo, Bárbara Parra,Paz Tapia, Jaime Lagos, Loredana Arata, Alejandra Acevedo, Winston Andrade, Gabriel Leal, Carolina Tambley, Patricia Bustos, Rodrigo Fasce, Jorge Fernandez
EPI_ISL_445364	HOSPITAL EL CARMEN DR.LUIS VALENTIN F.	Instituto de Salud Publica de Chile	Andrés E Castillo, Bárbara Parra,Paz Tapia, Jaime Lagos, Loredana Arata, Alejandra Acevedo, Winston Andrade, Gabriel Leal, Carolina Tambley, Patricia Bustos, Rodrigo Fasce, Jorge Fernandez
EPI_ISL_445365, EPI_ISL_445366	HOSPITAL DR.SOTERO DEL RIO	Instituto de Salud Publica de Chile	Andrés E Castillo, Bárbara Parra,Paz Tapia, Jaime Lagos, Loredana Arata, Alejandra Acevedo, Winston Andrade, Gabriel Leal, Carolina Tambley, Patricia Bustos, Rodrigo Fasce, Jorge Fernandez
EPI_ISL_445367	ASISTENCIA PUBLICA DR.ALEJANDRO DEL RIO	Instituto de Salud Publica de Chile	Andrés E Castillo, Bárbara Parra,Paz Tapia, Jaime Lagos, Loredana Arata, Alejandra Acevedo, Winston Andrade, Gabriel Leal, Carolina Tambley, Patricia Bustos, Rodrigo Fasce, Jorge Fernandez
EPI_ISL_445368	HOSPITAL DEL PROFESOR	Instituto de Salud Publica de Chile	Andrés E Castillo, Bárbara Parra,Paz Tapia, Jaime Lagos, Loredana Arata, Alejandra Acevedo, Winston Andrade, Gabriel Leal, Carolina Tambley, Patricia Bustos, Rodrigo Fasce, Jorge Fernandez
EPI_ISL_445369, EPI_ISL_445370	HOSPITAL DE CARABINEROS	Instituto de Salud Publica de Chile	Andrés E Castillo, Bárbara Parra,Paz Tapia, Jaime Lagos, Loredana Arata, Alejandra Acevedo, Winston Andrade, Gabriel Leal, Carolina Tambley, Patricia Bustos, Rodrigo Fasce, Jorge Fernandez
EPI_ISL_445371	HOSPITAL DR.SOTERO DEL RIO	Instituto de Salud Publica de Chile	Andrés E Castillo, Bárbara Parra,Paz Tapia, Jaime Lagos, Loredana Arata, Alejandra Acevedo, Winston Andrade, Gabriel Leal, Carolina Tambley, Patricia Bustos, Rodrigo Fasce, Jorge Fernandez
EPI_ISL_445372	HOSPITAL FF.AA. "CIRUJANO C. GUZMAN	Instituto de Salud Publica de Chile	Andrés E Castillo, Bárbara Parra,Paz Tapia, Jaime Lagos, Loredana Arata, Alejandra Acevedo, Winston Andrade, Gabriel Leal, Carolina Tambley, Patricia Bustos, Rodrigo Fasce, Jorge Fernandez
EPI_ISL_445373, EPI_ISL_445374, EPI_ISL_445375, EPI_ISL_445376, EPI_ISL_445377	HOSPITAL SAN JUAN DE DIOS	Instituto de Salud Publica de Chile	Andrés E Castillo, Bárbara Parra,Paz Tapia, Jaime Lagos, Loredana Arata, Alejandra Acevedo, Winston Andrade, Gabriel Leal, Carolina Tambley, Patricia Bustos, Rodrigo Fasce, Jorge Fernandez
EPI_ISL_445378	HOSPITAL DE BULNES	Instituto de Salud Publica de Chile	Andrés E Castillo, Bárbara Parra,Paz Tapia, Jaime Lagos, Loredana Arata, Alejandra Acevedo, Winston Andrade, Gabriel Leal, Carolina Tambley, Patricia Bustos, Rodrigo Fasce, Jorge Fernandez
EPI_ISL_445379	IMALAB- HOSPITAL FACH	Instituto de Salud Publica de Chile	Andrés E Castillo, Bárbara Parra,Paz Tapia, Jaime Lagos, Loredana Arata, Alejandra Acevedo, Winston Andrade, Gabriel Leal, Carolina Tambley, Patricia Bustos, Rodrigo Fasce, Jorge Fernandez
EPI_ISL_447119	HOSPITAL DR.HERNAN HENRIQUEZ ARAVENA	Instituto de Salud Publica de Chile	Andrés E Castillo, Bárbara Parra,Paz Tapia, Jaime Lagos, Loredana Arata, Alejandra Acevedo, Winston Andrade, Gabriel Leal, Carolina Tambley, Patricia Bustos, Rodrigo Fasce, Jorge Fernandez
EPI_ISL_449800	HOSPITAL SAN JUAN DE DIOS	Instituto de Salud Publica de Chile	Andrés E Castillo, Bárbara Parra,Paz Tapia, Jaime Lagos, Loredana Arata, Alejandra Acevedo, Winston Andrade, Gabriel Leal, Carolina Tambley, Patricia Bustos, Rodrigo Fasce, Jorge Fernandez
EPI_ISL_459856, EPI_ISL_459857, EPI_ISL_459858, EPI_ISL_459859, EPI_ISL_459860, EPI_ISL_459861, EPI_ISL_459862, EPI_ISL_459863, EPI_ISL_459864	Center for Genome Regulation (CRG)	Center for Mathematical Modeling and Center for Genome Regulation. Santiago, Chile	Gaete A, Travisany D, Palma R, Urra C, Varas M, Allende ML, Maass A, González M.
EPI_ISL_468747, EPI_ISL_468748, EPI_ISL_468749, EPI_ISL_468750, EPI_ISL_468751	Facultad de Medicina UC	Center for Mathematical Modeling and Center for Genome Regulation. Santiago, Chile	Gaete A, Travisany D, Palma R, Urra C, Varas M, Allende ML, Maass A, González M, Ferres M.
EPI_ISL_468752	Center for Genome Regulation (CRG)	Center for Mathematical Modeling and Center for Genome Regulation. Santiago, Chile	Gaete A, Travisany D, Palma R, Urra C, Varas M, Allende ML, Maass A, González M.
EPI_ISL_468753, EPI_ISL_468754, EPI_ISL_468755, EPI_ISL_468756, EPI_ISL_468757, EPI_ISL_468758, EPI_ISL_468759	Laboratorio de Biologia Molecular, Facultad de Medicina, Universidad de Atacama	Center for Mathematical Modeling and Center for Genome Regulation. Santiago, Chile	Gaete A, Travisany D, Palma R, Urra C, Varas M, Allende ML, Maass A, González M, C Echeverria
EPI_ISL_468760	Center for Genome Regulation (CRG)	Center for Mathematical Modeling and Center for Genome Regulation. Santiago, Chile	Gaete A, Travisany D, Palma R, Urra C, Varas M, Allende ML, Maass A, González M.
